# The protective power of connection: a proposed conceptual model of social supports in the context of youth adversity, disrupted attachment, and trauma symptoms

**DOI:** 10.3389/fpsyg.2026.1708589

**Published:** 2026-02-27

**Authors:** Dana M. Sox, Nancy L. Deutsch, Patricia A. Jennings, Helen H. Min

**Affiliations:** 1 School of Education and Human Development, University of Virginia, Charlottesville, VA, United States; 2 School of Law, University of Virginia, Charlottesville, VA, United States

**Keywords:** adversity, attachment, Bronfenbrenner Ecological Systems Theory, social support, trauma symptoms

## Abstract

Youths’ exposure to adversity is a significant contributor to the current pediatric mental health crisis. Social support is believed to have the power to promote positive developmental outcomes for young people, even helping to mitigate the risks associated with adversity experiences. However, both internal and environmental ecological systems shape how youth perceive and access the social supports available within their network. This paper draws upon various foundational theorists to propose a theoretically and empirically supported conceptual model that works to disentangle these nuanced and complicated factors influencing young people’s perceptions of social support and related developmental outcomes. This paper also discusses future implications for practice.

## Introduction

Childhood adversity is unfortunately common. According to the 2022–2023 National Survey of Children’s Health, approximately 39% of American students have encountered one or more Adverse Childhood Experiences (ACEs; Child and Adolescent Health Measurement Initiative, 2022–2023). Researchers first used the Adverse Childhood Experiences (ACEs) scale to examine the association between adverse childhood experiences and negative outcomes in adulthood (Felitti et al., 1998). The adverse experiences examined were based on two categories: abuse (psychological, physical, or sexual) and household dysfunction (substance abuse, mental illness, mother’s abuse, and criminal behavior in the household). The initial study of ACEs revealed a high prevalence of adverse experiences among the sample (half of the participants had at least one ACE) and an association between higher ACE scores and a higher risk of negative outcomes (Felitti et al., 1998). Since that original study, marginalized children (i.e., Black, Latino/Latina, below the federal poverty line, rural, and involved in the juvenile justice system) have been shown to have a higher likelihood of higher ACE exposures than comparison groups (Baglivio et al., 2014; Bruner, 2017; Crouch et al., 2020; Merrick et al., 2018). Further, these adversity experiences have been shown to negatively influence individuals socially, behaviorally, and academically from childhood into adulthood (Anda et al., 2005; Daines et al., 2021; Garrido et al., 2017; Lansford et al., 2002; Merrick et al., 2017; Weber Ku et al., 2021).

However, growing recognition that protective experiences can co-occur with adversity has shifted research attention toward understanding resilience mechanisms. The ICARE (Intergenerational and Cumulative Adverse and Resilient Experiences) model emphasizes that protective experiences, including supportive relationships, can buffer against risks associated with adversity (Hays-Grudo et al., 2021). Building on this foundation, scholars have increasingly examined how social support operates as a protective factor, though questions remain about the specific mechanisms and conditions under which support is most effective.

Within this paper, adverse childhood experiences and adversity exposures refer to a particular event and experience, whereas trauma symptoms are defined using the Substance Abuse and Mental Health Services Administration (SAMHSA) definition of an individual trauma. According to SAMHSA, an individual trauma is one resulting “from an event, series of events, or set of circumstances that is experienced by an individual as physically or emotionally harmful or life-threatening and that has lasting adverse effects on the individual’s functioning and mental, physical, social, emotional, or spiritual well-being” (SAMHSA's Concept of Trauma and Guidance for a Trauma-Informed Approach, 2014). Therefore, within the current paper, trauma symptoms involve an adverse event such as physical or sexual abuse, neglect, experiencing or witnessing domestic, community, or school violence, and natural or manmade disasters (Cavanaugh, 2016; Rumsey and Milsom, 2019) that result in lasting adverse effects on the individual. Importantly, while children may experience the same adverse event, they can exhibit different trauma responses, highlighting the role of individual variability and protective factors in shaping developmental outcomes. Similarly to adversity experiences, negative outcomes for youth with trauma symptoms are highly documented (e.g., decrement in IQ and reading achievement, increased likelihood of repeating a grade, lacking focus, demonstrating aggressive behavior, existing in states of hyperarousal, and exhibiting difficulties deciphering situations; Alvarez, 2020; Bethell et al., 2014; Delaney-Black et al., 2002; Van der Kolk, 2015).

Past empirical and theoretical literature has thoroughly documented the connections between adversity exposure, disrupted attachment to parenting adults, and a range of negative long-term psychological and social development outcomes, including trauma symptoms (e.g., Cushing et al., 2024; Wright et al., 2023). This same body of work has also highlighted the protective role of strong, positive attachments to parenting adults, which can buffer against the negative influence of adversity exposures and promote better developmental trajectories for young people (e.g., Barazzone et al., 2019; Darling Rasmussen et al., 2019). However, less is known about whether and how other forms of social support (e.g., teachers, program leaders, coaches, and peers) can bolster young people’s perceived sense of support and serve a similar protective role, helping to mitigate the risks associated with adversity. Furthermore, questions remain about the specific conditions that provide young people with developmentally supportive social supports and whether they have the necessary tools to access social supports available to them. For example, one might consider teachers a possible source of social support for a young person, but are educators able to help their students engage with and activate social support opportunities? Do students know that teachers may be available for support? Additionally, while research has identified various protective factors, less work has determined how these factors operate across multiple ecological levels simultaneously or how barriers at one level interact with protective mechanisms at another.

This paper presents a conceptual model, developed using a theory-building approach, to advance understanding of how perceived social support functions as a protective factor for youth exposed to adversity. Following Lynham’s (2002) Theory-Research Cycle framework, we draw on existing empirical evidence to propose theoretical relationships among constructs at individual, microsystem, mesosystem, exosystem, and macrosystem levels, articulating how social support operates, what limits its effectiveness, and how barriers can be overcome through intentional intervention.

## Theoretical contributions

Bronfenbrenner’s Ecological Systems Theory (Bronfenbrenner, 1977, 1979) posits that as an individual develops, they are influenced both biologically and social–emotionally by their immediate environment(s) (i.e., family, school, and community) as well as larger political, cultural, societal, and environmental structures. The process-person-context-time (PPCT; Williams and Ceci, 1997) model was later developed from Bronfenbrenner’s bioecological theory and emphasized the dynamic interplay between process (interactions with the environment), person (individual characteristics), context (environmental systems), and time (temporal influences across the life course). Within this framework, social support is understood as ecologically distributed across systems in a young person’s environment.

At the microsystem level, young people can access different levels and types of support through various close relationships. These multiple sources of support can have a cumulative, reinforcing influence on youths’ developmental outcomes. In particular, rather than youth relying solely on a singular relationship (e.g., parent or teacher), youth can find differentiated support through a network of interconnected relationships that work together to address the diverse and ever-evolving needs of a young person (e.g., Varga and Zaff, 2018). This layered, relational approach emphasizes the holistic nature of human connection and the value of distributed care in supporting youth thriving and well-being. For example, macrosystem-level factors have the potential to directly influence activities and outcomes at a microsystem level. In particular, when teachers are supported with adequate time and resources, they are better positioned to build meaningful relationships with students within their classrooms; conversely, limited time and resources can put excess strain on educators, making them feel as if they do not have time allocated in their day to engage meaningfully with students.

The Developmental Assets Framework (Roehlkepartain and Blyth, 2019) compliments Bronfenbrenner’s Ecological Systems Theory by identifying specific assets both internal (i.e., academic engagement, positive identity, positive values, and social competencies) and external (i.e., support, mattering and belonging, and boundaries) to an individual that contribute to healthy developmental trajectories (Syvertsen et al., 2021). While Bronfenbrenner’s theory explains where and how development occurs within various systems, the Developmental Assets Framework describes the specific resources that promote healthy development across different contexts.

The Developmental Assets Framework is further complemented by the ICARE (Intergenerational and Cumulative Adverse and Resilient Experiences) model, which focuses specifically on the role of developmental supports in relation to adversity experiences (Hays-Grudo et al., 2021). The ICARE model acknowledges that protective and compensatory experiences (PACEs), such as strong relationships and ample resources (e.g., a safe and clean home with food), can buffer against adverse experiences and provide a framework for understanding the biobehavioral adaptations that link adverse experiences with maladaptive outcomes later in life (Hays-Grudo et al., 2021). These foundational theories are used in this paper to ground our understanding of the systems and relationships that influence development, as well as the particular assets, including social supports, that foster positive developmental outcomes.

### Approach to theory development

This paper employs Lynham’s (2002) Theory-Research Cycle approach to theory development, which conceptualizes theory building as an iterative process involving five phases: conceptual development, operationalization, confirmation or disconfirmation (i.e., empirical testing), application, and ongoing refinement and development. Consistent with theory development methodology (Lynham, 2002), we present our postulations about how social support operates at each ecological level, supported by existing empirical evidence, and then identify critical gaps requiring future empirical testing. We organize these propositions into three thematic sections: (1) Protective Mechanisms, examining how social support functions to buffer adversity effects across individual, microsystem, mesosystem, exosystem, and macrosystem levels; (2) Barriers and Limitations, exploring factors that impede social support effectiveness at each level; and (3) Intervention Pathways, proposing mechanisms through which barriers can be overcome and protective functions strengthened.

## Proposed conceptual model

**Figure 1:**
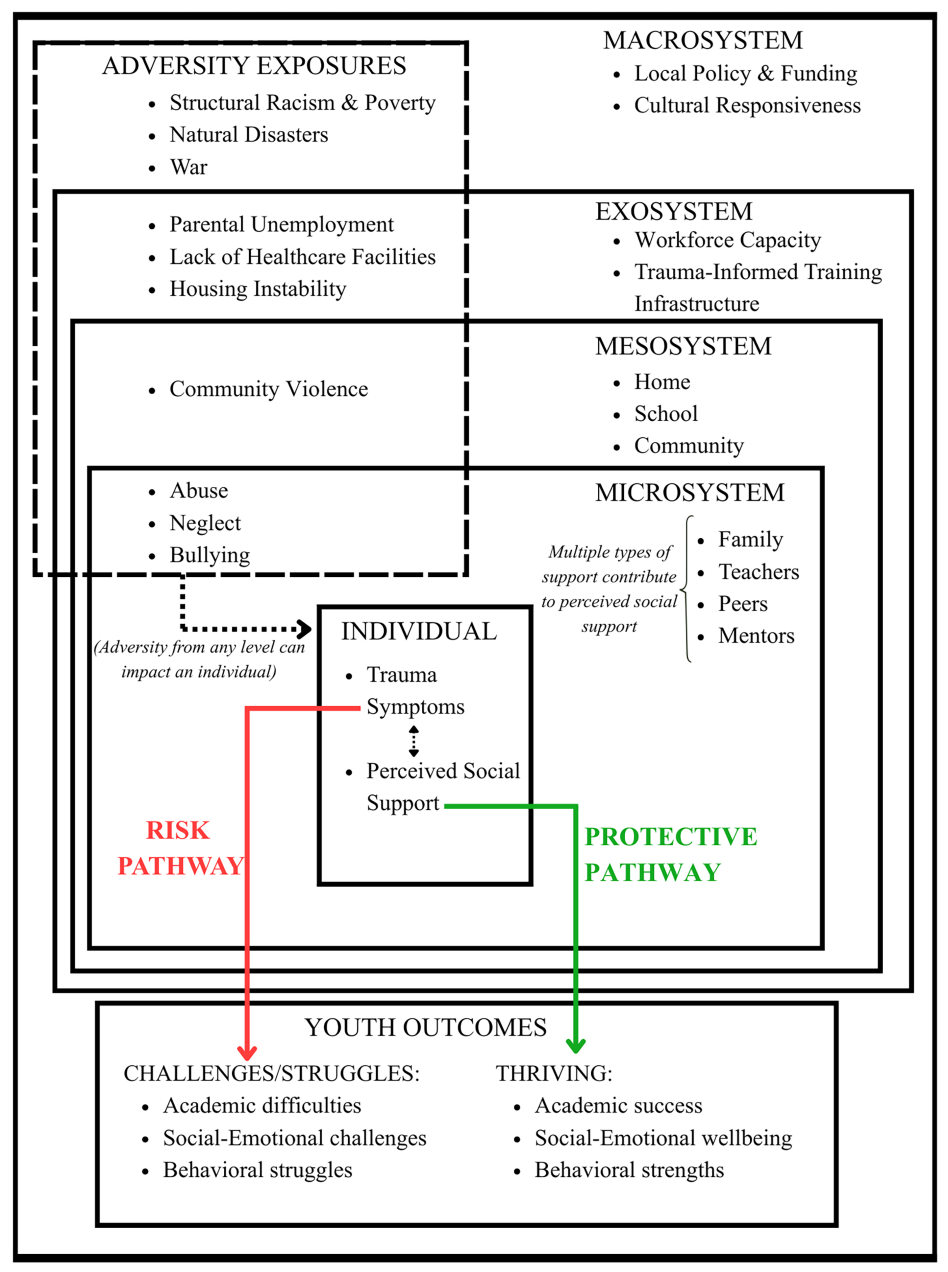
Ecological model of perceived social support as a protective factor.

Figure 1 presents an ecological model illustrating how perceived social support protects youth exposed to adversity. The model shows that adversity can originate at multiple ecological levels (macrosystem, exosystem, mesosystem, microsystem) and may lead to trauma symptoms, though responses vary individually. Two developmental pathways may emerge: a risk pathway (red) from trauma symptoms to developmental challenges, and a protective pathway (green) from perceived social support to thriving outcomes. These pathways interact bidirectionally, with trauma symptoms and perceived support mutually influencing each other. For example, trauma symptoms such as emotional dysregulation or withdrawal may influence a youth’s ability to recognize, seek, or trust available support, thereby reducing their perception of the strength and availability of social supports. Conversely, high perceived social support can protect against the risk associated with adversity experiences, reducing negative symptomatology and supporting positive developmental outcomes. Multiple microsystem support sources (family, teachers, peers, mentors) contribute to perceived support, which is strengthened by mesosystem coordination and enabled by exosystem conditions and macrosystem policies and resources. The model emphasizes that subjective perceptions of support, rather than its mere presence, serve as the key protective mechanism.

While this model draws on established frameworks such as Bronfenbrenner’s Ecological Systems Theory, Developmental Assets Framework, and the ICARE model, it also makes several theoretical contributions. First, it makes an explicit distinction between adversity exposure and trauma symptoms. Unlike models that treat these constructs interchangeably, we theorize trauma symptoms as a conditional outcome of adversity that shapes and is shaped by perceived social support. This distinction creates new intervention targets beyond reducing adversity exposure and conceptualizing trauma symptoms as an intervention outcome. Furthermore, we propose that trauma symptoms and perceived social support mutually influence one another over time, suggesting a bidirectional model rather than a unidirectional one in which social support simply buffers the risks associated with adversity. Finally, unlike models that position adversity experiences exclusively at the microsystem level, we theorize that adversity can originate at any ecological level, which requires consideration of multilevel interventions.

## Functional constructs: how social support protects across ecological levels

We begin by examining how social support acts as a protective mechanism across ecological levels. The conceptual framework proposes that perceived social support serves as a protective mechanism that buffers against the risk pathway from trauma symptoms to challenging developmental outcomes. At each ecological level, we present our theoretical postulations on how social supports function as protective factors, followed by empirical evidence supporting these propositions. Importantly, the framework and this section emphasize that protective mechanisms at each level are inextricably interconnected. For example, systemic policies can shape an individual’s experiences, while an individual’s experiences and communities’ needs can drive systemic policies.

### Individual-level protective mechanisms

At the individual level, we postulate that several internal developmental assets and personality traits function as protective factors, enabling young people to perceive, access, and benefit from available social support systems. Specifically, internal developmental assets such as emotional regulation, social competencies, and positive values equip youth with the interpersonal skills necessary to initiate and maintain supportive relationships (Roehlkepartain and Blyth, 2019). Similarly, certain personality traits (i.e., extraversion, conscientiousness, agreeableness, and openness to experience) have been empirically linked to greater perceived availability of social support. A meta-analysis of 72 studies representing 37,678 participants found that higher levels of these traits, along with lower neuroticism, were significantly associated with enhanced perceived social support (Barańczuk, 2019).

Beyond facilitating relationship formation, we propose that positive perceived social support itself functions as an internal cognitive and emotional resource. This perceived support, distinct from the mere presence of supportive others, serves as a psychological buffer that protects against the negative impacts of adversity. Research demonstrates that perceived social support is associated with reduced depression symptoms (Rueger et al., 2016), decreased posttraumatic stress symptoms (Allen et al., 2021; Xiong et al., 2022), and enhanced overall quality of life (Amedu et al., 2025). Furthermore, this evidence supports our framework’s emphasis on the perception of social support as the key protective mechanism.

Additionally, certain internal protective factors such as self-efficacy, optimism, hope, internal locus of control, and self-esteem have been shown to buffer against adverse events by influencing how individuals appraise stressors, engage in coping strategies, and maintain psychological resilience in the face of challenges (Agbaria and Abu Mokh, 2022; Frankham et al., 2020; Kaniasty et al., 2025; Pruessner et al., 2005). For instance, research indicates that internal locus of control and self-esteem are associated with more effective coping mechanisms and greater life satisfaction, even when individuals encounter negative life events (Pruessner et al., 2005). These internal protective factors can be strengthened through supportive relationships, which have been empirically shown to improve interpersonal assets, such as self-esteem (Harris and Orth, 2020).

Importantly, individual variability in these protective traits helps explain why young people respond differently to similar adverse experiences. According to Bronfenbrenner’s Ecological Systems Theory, the individual at the core of the model emphasizes the bidirectional interaction between an individual and their environment, such that individual characteristics and traits directly influence how an individual interacts with their environment and how the environment responds (Bronfenbrenner, 1979). These characteristics influence not only how adversity is experienced but also how social support is perceived and utilized. For example, an individual can perceive and internalize adverse experiences within their environment in different ways (e.g., Punamäki et al., 2006). Even siblings exposed to the same environmental stressors can exhibit markedly different outcomes. A comparative study of 38 sibling pairs (ages 6–17) exposed to a natural disaster found that PTSD symptoms were uncorrelated between siblings, with within-family differences (between siblings) comparable to those observed between randomly selected, unrelated children (Nygaard et al., 2010). This finding underscores a central postulate of our theory: individual characteristics, including personality traits, developmental assets, and the capacity to perceive social support, function as critical moderators that shape how adversity exposures translate into developmental outcomes.

### Microsystem-level protective mechanisms

The microsystem refers to the entities found in a young person’s immediate environment, including family members, teachers, coaches, and peers (Bronfenbrenner, 1977, 1979). At the microsystem level, we postulate that perceived social support can act as a protective factor for youth facing adversity, supporting their psychological outcomes and overall quality of life (e.g., Amedu et al., 2025).

A strong and positive attachment to one’s parenting adult can be a source of perceived social support for young people and serve as a protective factor, helping mitigate the risks associated with exposure to adversity (e.g., Pekel et al., 2018; Roehlkepartain et al., 2017). Research also suggests that different sources of support within the microsystem may contribute uniquely to specific developmental outcomes. Using structural equation modeling (SEM), a study of 733 adolescents (grades 7–8) without noted adversity found that perceived teacher support was positively associated with students’ self-worth (*ꞵ* = 0.20) and physical well-being (*ꞵ* = 0.22), peer support with psychological well-being (*ꞵ* = 0.25), and parental support with all three (i.e., self-worth (*ꞵ* = 0.32), physical well-being (*ꞵ* = 0.20), and psychological well-being (*ꞵ* = 0.37); Hoferichter et al., 2021). This finding suggests that individuals may receive differing levels and types of support from different individuals within their microsystem, with parental support being the most influential.

Additionally, at a neurobiological level, perceived social support may be linked with more adaptive processing of stress and threat. A cross-sectional study of 55 children (ages 7–16) found that greater perceived support was positively associated with more efficient neural processing of threat stimuli in youth exposed to adversity (Wymbs et al., 2020). Furthermore, research has shown that people perceive less pain when they receive a physical stimulus while holding the hand of someone they love (Coan et al., 2006). Taken together, these findings suggest a connection between perceived social support in the microsystem and protective factors for youth facing adversity.

### Mesosytem-level protective mechanisms

Bronfenbrenner’s Ecological Systems Theory emphasizes the mesosystem (i.e., the network of interactions between a young person’s immediate environments, such as home and school) as a critical factor in developmental trajectories (Bronfenbrenner, 1977, 1979). At the mesosystem level, our conceptual model postulates that social support across multiple environments, such as both family and school contexts, plays an important role in mitigating risk and fostering youth well-being (Amedu et al., 2025; Belea and Calauz, 2019; Yule et al., 2019). This is also aligned with Hays-Grudo and Morris’ (2020) and Hays-Grudo et al.’ (2021) work, which emphasizes that protective experiences and adverse experiences can operate simultaneously. Like the ICARE model (Hays-Grudo et al., 2021), our framework positions social support not merely as the absence of adversity, but as an active developmental resource that can co-occur with adversity and promote positive developmental pathways despite risk.

Young people can access different types and levels of support through various close relationships. These multiple sources of support can have a cumulative, reinforcing influence on youths’ developmental outcomes. In particular, rather than youth relying solely on a singular relationship (e.g., parent or teacher), youth can find differentiated support through a network of interconnected relationships across their microsystems that work together to address the diverse and ever-evolving needs of a young person (e.g., Varga and Zaff, 2018). This layered, relational approach emphasizes the holistic nature of human connection and the value of distributed care in supporting youth thriving and well-being.

Some evidence suggests that the combined supportive efforts of families and teachers can reduce depression symptoms in adolescents (e.g., Rueger et al., 2014). For example, a longitudinal study of 1,452 Australian students (ages 11–16) found that perceived family and teacher support predicted fewer depressive symptoms 1 year later (Pössel et al., 2018). Furthermore, these interactions between microsystems have the potential to benefit more than just the youth. For example, interviews with 20 foster families revealed the importance of having multiple systems to rely on for support, as well as learning to navigate these various systems by gathering information from individuals within their networks (Piel et al., 2017). Similarly, in a study of 95 youth involved in the juvenile justice system, community networks and support, including extended family, school, recreation activities, faith-based groups, work, and neighbors, were related to better family functioning and positive peer relations, and, indirectly, to youth self-reliance (Smith et al., 2016). Thus, the interactions that form the mesosystem serve as an important conduit through which families and youth access resources, supports, and information that help protect youth from risks associated with adversity experiences.

### Exosystem and macrosystem-level protective mechanisms

The exosystem and macrosystem refer to the broader systems and cultural contexts within which a young person and their environment are embedded (Bronfenbrenner, 1977, 1979). The exosystem encompasses organizations and services that indirectly affect youth, while the macrosystem includes broader environmental forces, including collective cultural strengths and systemic racism. At these levels, our conceptual model postulates that institutions, culture, and politics can significantly influence how a young person accesses and benefits from social support systems (e.g., Keles and Oppedal, 2022). Although these systems are well recognized, developmental research has historically focused on examining factors in the microsystem, which are proximally closer to an individual child (Onnie Rogers et al., 2021). Onnie Rogers et al. (2021) proposed the use of *m(ai)cro* as a way to shift the start and focus of the story to be the macrosystem, acknowledging the influence macrosystem-level factors, such as structural racism, have on the other ecological levels. Because empirical studies on protective social supports found at these levels are scant, for this section, we use an example of how cultural responsiveness can enhance social support as a way to indirectly speak to the potential and power of exosystem and macrosystem-level supports.

Incorporating cultural responsiveness into socially supportive spaces commonly used by young people can enhance their perceptions of social support by creating environments that feel more welcoming, inclusive, and affirming. In particular, research demonstrates that youth from marginalized communities are more likely to engage with and benefit from programs when cultural responsiveness is prioritized. For example, a study of 134 Latine youth who participated in an afterschool math enrichment activity showed a positive association between adolescents’ perceptions of diverse teaching practices and increased feelings of autonomy, competence, and relatedness (Wycoff et al., 2025). Similarly, a study of 1,930 African American youth in a mentoring program found that cultural orientation, Africentric values, and cultural orientation predicted positive youth development outcomes (i.e., future orientation, prosocial behavior, political and community civic-mindedness, and social justice and equality civic-mindedness; Grills et al., 2016).

Organizational structures and practices at the exosystem level shape the cultural responsiveness of programs in the youth’s microsystem. Simpkins and Riggs (2014) provide a framework for building cultural competence in afterschool programs, emphasizing alignment of organizational structures, policies, and staff practices with community needs (e.g., hiring diverse staff, providing multilingual communication, and ensuring staff’s cultural knowledge of the youth and families they serve). Further supporting the importance of cultural responsiveness, some evidence suggests the importance of adult-youth racial-ethnic matching, an organizational-level practice, in youth mentoring programs. For example, a study of 82,224 mentor and mentee matches found that matches with reported shared racial or ethnic identities were less likely to experience early match termination (Lyons and Edwards, 2022). However, other literature suggests that mentoring racial-ethnic matching is not a significant predictor of relationship quality (Koide et al., 2024).

Local policy and funding allocation at the macrosystem level play a direct role in supporting or hindering organizations’ ability to build cultural competence and workforce capacity. Local policymakers determine the level of funding allocated to youth-serving programs, schools, and community-based supports, which directly influences organizational capacity to hire diverse staff, provide culturally responsive programming, and create supportive contexts. Research also demonstrates clear associations between funding levels and outcomes. For example, an analysis of 100 schools found positive correlations between investment in teacher salaries and professional development and higher student achievement (Sheng, 2023).

## Limitations: barriers to support across ecological levels

While systems of social support can operate as protective mechanisms across ecological levels, strengthening the protective pathway illustrated in our conceptual framework (Figure 1), multiple factors can limit their effectiveness. These barriers operate in three key ways that undermine the framework’s protective mechanisms: (1) they impair youth’s capacity to perceive available support, (2) disrupt the quality or availability of microsystem relationships that provide social support, and (3) prevent coordination across contexts and adequate allocation of resources. Furthermore, barriers at one level often cascade to other levels. The following section examines these barriers at each ecological level, illustrating how they weaken the protective pathway and, in some cases, strengthen the risk pathway from adversity exposures to developmental challenges.

### Individual-level barriers

At the individual level, several factors can impede young people’s capacity to perceive, access, and benefit from available social support. These barriers operate through three primary mechanisms: intergenerational trauma and epigenetic influences, trauma-related disruptions to perceived support, personality and character traits that influence relationship formation, and neurobiological changes that influence the processing of social information.

Intergenerational trauma and epigenetic influences may shape individual responses to adversity and the capacity to perceive support. In particular, parental trauma exposure can influence offspring stress reactivity through epigenetic mechanisms, which have been shown to influence brain development and disease risk (Chan et al., 2018). Additionally, intergenerational patterns of attachment and relational schemas may be transmitted across generations, influencing youths’ expectations about adult availability and responsiveness (Hays-Grudo and Morris, 2020; Smrtnik Vitulić et al., 2023). Thus, biological and relational inheritances represent individual-level factors that may moderate the relationship between available support and perceived support.

When youth experience trauma symptoms (e.g., heightened states of arousal, difficulties with emotional regulation, and altered perceptions of threat), their ability to recognize or trust supportive relationships may be compromised. Evidence suggests that higher levels of posttraumatic stress symptoms have been found to be associated with lower levels of perceived social support (Birkeland et al., 2020), creating a problematic feedback loop wherein those most in need of support may be less able to access it. This dynamic operates through multiple pathways. Trauma symptoms may increase vigilance for threat cues while decreasing sensitivity to safety cues, making it difficult for youth to accurately assess when adults are trustworthy and supportive. For example, a study of 26 adversity-exposed youth (ages 17–24) found that these young people responded with heightened fear even to safe cues, making it difficult for them to distinguish between threatening and safe situations during learning tasks (Qiao et al., 2025). Adversity exposure can also produce neurobiological changes that disrupt the processing of social and threat-related cues, further impairing access to support. A cross-sectional study of 55 children (ages 7–16) found that greater perceived support was positively associated with more efficient neural processing of threat stimuli in youth exposed to adversity (Wymbs et al., 2020). This finding suggests that the absence of perceived support may correspond with less efficient neural processing, potentially creating a neurobiological mechanism through which low perceived support perpetuates difficulty recognizing safety and trustworthiness in relationships. Finally, trauma-related emotional dysregulation may strain relationships, inadvertently pushing away potential sources of support (e.g., Cox et al., 2017).

Past studies have also linked personality traits (e.g., conscientiousness and extraversion) to perceived social support, suggesting that certain personality traits may facilitate the development and maintenance of supportive relationships (Barańczuk, 2019; Cukrowicz et al., 2008; Lakey and Scoboria, 2005). However, this raises important questions about equity. In particular, if certain traits naturally elicit greater support from adults, youth lacking these traits may receive less support regardless of their needs. Furthermore, this pattern suggests that commonly used measures of perceived social support may, in part, reflect underlying individual characteristics.

### Microsystem-level barriers

At the microsystem level, disruptions to foundational relationships can severely limit the protective capacity of social support systems. Childhood adversity, particularly when derived from parenting adults, can disrupt the natural attachment that develops between youth and their parenting adults. This disruption, or broken attachment, has been linked to negative long-term psychological and social developmental outcomes (e.g., Joubert et al., 2012). Family-based adversity may impair young people’s ability to form trusting relationships with other adults across their microsystem.

Importantly, the quality of adult–youth relationships is highly variable, particularly among youth with histories of adversity. While some research finds that supportive parental relationships can help youth regulate emotions and navigate stressors more effectively (Pekel et al., 2018; Roehlkepartain et al., 2017), other work suggests that youth exposed to adversity may struggle to build trusting relationships with adults (Poon et al., 2021) and often report lower-quality connections (Scales et al., 2020). This may be in part related to the association between some forms of childhood adversity (e.g., abuse, neglect, parental incarceration) and disrupted attachment to parenting adults. Additionally, the buffering effect of family support may diminish in cases of more severe maltreatment, especially among girls and women, raising further questions about the limits of support within familial contexts (Evans et al., 2013).

Beyond family relationships, the capacity of other microsystem members, such as teachers, coaches, and mentors, to provide effective support may be constrained by insufficient preparation, resources, and time (see Macrosystem-Level Barriers for additional structural constraints). For example, a preassessment of 95 mental health professionals found that 68% of participants felt inadequately trained to assess trauma and 75% felt inadequately trained to treat it (Kumar et al., 2022). When adults lack the specialized skills, consistency, patience, and empathy needed to work with trauma-affected youth, their well-intentioned efforts to provide support may fall short.

### Mesosystem-level barriers

At the mesosystem level, fragmentation and lack of coordination across support systems can substantially diminish the protective effects of social support systems. While research demonstrates that support across multiple contexts (family, school, community) promotes youth well-being (Amedu et al., 2025; Belea and Calauz, 2019; Yule et al., 2019), interventions typically remain siloed within individual systems, and critical gaps persist in understanding how different support sources effectively interact with one another. A national study of 18,160 youth (ages 6–17 years) found that only 23.5% of caregivers reported communication between the child’s school and health care provider (Geffel et al., 2022). Additionally, the absence of shared frameworks compounds these coordination challenges. A qualitative study of 126 child-service providers found that they felt knowledgeable about their definitions of trauma-informed practices, even though there was substantial variation in their understanding and the application of these practices across participants (Donisch et al., 2016). Without intentional communication mechanisms and coordinated protocols, young people must navigate disconnected support systems independently, creating gaps in care and missing opportunities for the combined benefits of coordinated systems. For example, a youth might work with a school counselor on trauma coping strategies while simultaneously experiencing family-based triggers that the counselor does not know about, or a mentor might utilize approaches that contradict strategies taught in therapy, all of which can diminish the perceived strength and availability of social support.

### Exosystem and macrosystem-level barriers

At the exosystem and macrosystem level, structural inequities and policy failures create systemic barriers that limit equitable access to social support for young people. Areas with the greatest need for social support (e.g., high rates of community violence and poverty) may have the least access to these resources (e.g., Baker et al., 2021). This inverse relationship between need and resource availability deepens existing disparities and weakens the protective role of support systems for the most vulnerable youth. Multiple indicators reveal the scope of these structural limitations. A nationally representative survey of 31,055 randomly selected households in 2020 found an increase in unmet demand for afterschool programs; that is, for every child enrolled in an afterschool program, approximately three more are waiting to be admitted (Anda et al., 2005). This opportunity gap is further exacerbated by the cost of involvement in afterschool programs, extracurricular activities, and summer programs, whereby families in the highest income bracket, on average, report spending more than five times as much on afterschool activities as families in the lowest income bracket (Anda et al., 2005), effectively pricing out the young people who might benefit most from additional supportive contexts.

Furthermore, at the exosystem level, the shortage of school mental health professionals and staff represents another barrier. According to recent guidelines by the National Association of School Psychologists (NASP), the U.S. needs approximately 63,000 more school psychologists to meet the recommended ratio that ensures all students have access to the necessary school psychological services (Lipkin, 2023). This shortage leaves countless youth without access to specialized mental health support within their educational environments.

Beyond resource scarcity, the absence of culturally responsive practices in many youth-serving organizations creates environments that fail to feel welcoming, inclusive, or affirming for young people from diverse backgrounds. For example, in a study of 16 special education students in a low-income, ethnically diverse middle school, a majority of the students reported that their race and culture were not acknowledged by their teachers or taught in their classes, and students described racial violence and animosity as a challenge (Hughes et al., 2011). When organizational structures, policies, and staff practices do not align with the cultural values and needs of the communities they serve, young people’s perceptions of available support may be diminished regardless of adults’ intentions.

Finally, the limited integration of trauma-informed training across sectors at both the exosystem and macrosystem levels leaves many adults who interact with youth unprepared to recognize trauma responses or provide appropriate support. For example, despite serving youth with high levels of risk (e.g., low income, family stress, or marginalized racial backgrounds), mentoring programs often offer universal programming with limited mentor training and few specialized supports for youth, resulting in small overall effect sizes (Rhodes, 2020). Furthermore, although culturally responsive trauma-informed trainings are needed to support the development and retention of socially supportive relationships for young people, they are rarely integrated into trauma-informed interventions and trainings. A study of 20 community practitioners within a Latine community in Phoenix, Arizona, highlighted several barriers to effectively implementing culturally responsive trauma-informed services, including impoverished and isolated communities, mistrust in the system, a normalization of trauma, continuous exposure to adversity, and a lack of relevant training for staff and professionals (Wycoff et al., 2025). Without macrosystem-level policy mandates that establish training standards and allocate resources for ongoing professional development, these systemic gaps persist across sectors, diminishing the effectiveness of social supports.

## Promoting change: intervention pathways across ecological levels

Despite the perceived social support barriers that can be more prevalent among individuals exposed to adversity, evidence suggests multiple pathways to promote change that address these barriers. Our conceptual framework (Figure 1) proposes that perceived social support operates through a protective pathway that buffers against the risk pathway from trauma symptoms to developmental challenges. The framework emphasizes three key principles that guide intervention design: (1) perceived support serves as the proximal protective mechanism, not simply the presence of support systems, (2) trauma symptoms and perceived support interact bidirectionally, (3) protective factors at lower ecological levels (i.e., individual, and microsystem) depend, at least in part, on enabling conditions at higher levels (e.g., coordination across social supports in the mesosystem, and macrosystem-level policies). The following section describes intervention opportunities at each ecological level.

### Individual-level interventions

At the individual level, we postulate that youth can develop skills to expand and strengthen their social networks, particularly when given intentional opportunities and guidance. Skills-based interventions have the capacity to enhance youth’s ability to perceive and access support. For example, an intervention for first-generation college students found that although young people enter college with different norms and expectations for higher education, skills-based workshops focused on social capital-building improved students’ self-advocacy and academic outcomes (Schwartz et al., 2018). Additionally, skills-based interventions can help enhance youth’s capacity to perceive and access available supports, thereby strengthening the protective pathway.

Trauma-focused interventions that increase perceived support have been shown to lead to decreased trauma symptoms, supporting the proposed bidirectional relationship between trauma and social support. A longitudinal, quantitative study examined perceived social support and posttraumatic stress symptoms (PTSS) in 156 participants (10–18 years old), randomized to receive either trauma-focused cognitive behavioral therapy (TF-CBT) or therapy as usual (Birkeland et al., 2020). Over 18 months following treatment, self-reported surveys were used to track changes. Using latent growth curve modeling, the study found that increases in perceived support during trauma-focused therapy were associated with decreases in posttraumatic stress symptoms, underscoring the potential dynamic role of social support in trauma recovery. This finding directly supports our conceptual framework’s proposition of a bidirectional relationship between trauma symptoms and perceived support (Figure 1), in that, when interventions successfully increase perceived support, trauma symptoms decrease, creating a virtuous cycle that decreases or even reverses the problematic feedback loop observed without intervention. Finally, the timing of interventions and the formation of relationships appear critical. One study found that meeting a natural mentor earlier in life was associated with higher levels of social–emotional well-being (Prowell and Williams, 2021), suggesting that social support interventions should begin as early as possible, as the timing of a mentoring relationship can shape its developmental outcomes.

### Microsystem-level interventions

At the microsystem level, we postulate that multiple pathways exist for strengthening supportive relationships and compensating when primary attachments are disrupted or unavailable. Two complementary frameworks guide intervention approaches: Rhodes’ model of mentoring (Rhodes et al., 2006), which emphasizes how mentoring relationships can improve parent–child relationships and provide missing support when primary attachments are compromised, and Webs of Support (Varga and Zaff, 2018), which positions youth’s broader network of relationships, rather than any single relationship, as the meaningful predictor of developmental outcomes. Evidence supports both approaches: a meta-analysis of 73 mentoring programs found moderate positive effects on social–emotional and behavioral outcomes (*g* = 0.21), with stronger program effects associated with programs serving young people with higher individual and environmental risk (DuBois et al., 2011). Similarly, a meta-analysis examining perceived social support from relational networks found moderate positive effects on well-being (*g* = 0.201; Chu et al., 2010). Additionally, parent-focused interventions that strengthen self-regulation skills and reduce dysregulated stress responses help disrupt the intergenerational transmission of adverse experiences (Hays-Grudo and Morris, 2020; Hays-Grudo et al., 2021). These findings support our framework’s proposition that multiple microsystem support sources, such as family, teachers, peers, and mentors, collectively contribute to perceived support by providing differentiated support across contexts, and indicate the importance of ensuring that those adults have the resources they need to provide effective support to youth.

Indeed, building effective relationships with trauma-affected youth requires specialized skills and training. A study of 455 mentor-mentee dyads found that mentors’ experiences (i.e., perception of program structure, supportive relationships, and opportunities for skill building) with Campus Connections (CC; a mentoring program for at-risk youth) were positively associated with mentoring relationship quality (Weiler et al., 2019). These findings suggest that equipping adults across microsystem contexts (e.g., teachers, mentors, coaches, program staff) with trauma-informed skills can enhance their capacity to form strong, responsive relationships with youth facing adversity. The role of the macrosystem in preparing adults to support youth with adversity exposure is critical, and discussed below.

### Mesosystem-level interventions

At the mesosystem level, we postulate that integrated efforts across schools, families, and community organizations may be key to strengthening the impact of social support for youth experiencing adversity. Scholars have emphasized the need for coordinated, cross-contextual support systems, particularly those that include accessible trauma-informed training across community sectors (Miller and Kolko, 2020; Osgood et al., 2010), suggesting that intentional coordination mechanisms can help overcome the limitations of siloed interventions. In a study on resilience, youth who experienced higher adversity and fewer supportive relationships demonstrated less adaptive capacity than those who had experienced less adversity and had strong supportive relationships with parents and other adults (Masten and Tellegen, 2012), highlighting the potential for multiple supportive relationships working together to improve adaptive outcomes. Our conceptual framework proposes that perceived support is strengthened by mesosystem coordination. When home, school, and community supports are fragmented, their protective capacity may be diluted; however, coordinated efforts may produce synergistic effects greater than the sum of individual supports.

Research highlights the importance of social support and community involvement in fostering positive outcomes for young people exposed to adversity. In particular, family support interventions can enhance adolescent coping by building social capital and resilience (Pinkerton and Dolan, 2007). Furthermore, for youth transitioning out of the child welfare system, continued family and community support is crucial for successful development (Collins, 2001). However, building cohesive communities for children and families is challenging and requires regular contact among community members and strong social networks (Moore, 2005). Comprehensive approaches that leverage social supports, community resources, and youth participation can create positive environments for young people’s growth and well-being.

### Exosystem and macrosystem-level interventions

At the exosystem and macrosystem level, we postulate that policy interventions and structural changes can create conditions that enable equitable access to high-quality social support for all young people. Our conceptual framework explicitly positions exosystem and macrosystem factors as supportive of microsystem relationship quality and mesosystem coordination. Thus, when macrosystem resources and policies are aligned with exosystem institutional capacity, mesosystem coordination efforts, and microsystem and individual-level needs, efforts across ecological levels can be bolstered. Four primary intervention pathways emerge from existing evidence at these levels: funding allocation, workforce development, youth engagement in policy development, and policies that promote cultural competence.

A central premise of this framework is that adults’ capacity to provide high-quality support depends on exosystem and macrosystem-level structural conditions. For example, a study of 135 teachers across 20 elementary schools found that small class sizes were associated with higher levels of teacher satisfaction, greater use of enriched classroom activities, and increased ability to respond to teacher needs (Price and Terry, 2008). Microsystem-level interventions must therefore be coupled with macrosystem-level policy decisions that provide adults with adequate time, resources, and ongoing support to build and sustain meaningful relationships.

Local and state policymakers at the macrosystem level determine funding levels for youth-serving programs, schools, and community-based supports. Evidence suggests that strategic funding increases produce measurable improvements in support quality and youth outcomes. For example, a quasi-experimental study examining increased state school funding in California found that a $1,000 increase in the average per-pupil spending was associated with improved academic achievement in math and reading in every grade level assessed, reduced likelihood of grade repetition, increased likelihood of high school graduation and college readiness, and decreased suspensions and expulsions (Johnson, 2023). Thus, increased funding to bolster interventions and programs that help increase young people’s social support networks can have direct and positive influences on developmental outcomes.

Addressing workforce shortages (Lipkin, 2023) and training gaps at the exosystem level requires systemic investment in recruitment, training infrastructures, and retention strategies at the macrosystem level. Beyond increasing workforce capacity, policy mandates for trauma-informed, culturally responsive training can improve support quality across sectors. A study of four schools that received Healthy Environments and Response to Trauma in Schools (HEARS) training found significant improvements in teachers’ knowledge and use of trauma-sensitive practices, staff perceptions of student engagement, and disciplinary referrals and suspensions (Dorado et al., 2016). Developing coordinated, cross-contextual support systems with accessible trauma-informed training across community sectors could create consistent standards while improving support effectiveness.

Communities are increasingly recognizing the value of engaging youth in shaping the policies and programs that affect them. A systematic review of 63 youth participatory action research (YPAR) studies found that the most common outcomes associated with YPAR participation were agency and leadership, academic or career, social, critical consciousness, interpersonal, and cognitive skills (Anyon et al., 2018). Policy mechanisms at the macrosystem level that institutionalize youth voice, such as youth advisory boards with decision-making authority, participatory budgeting processes, and youth representation in planning bodies, can enhance the effectiveness of support systems. For instance, an evaluation of youth-adult partnerships in afterschool program governance found that programs with formalized youth input structures demonstrated greater responsiveness to youth needs and higher youth satisfaction than adult-only governance models (Zeldin et al., 2013). These findings suggest that policies mandating meaningful youth engagement in program and policy development may enhance both the relevance and effectiveness of support systems.

Policy at the macrosystem level can mandate that youth organizations at the exosystem level implement practices and procedures that promote cultural competence. Building on frameworks like Simpkins and Riggs (2014), policies can require workforce diversity, multilingual communication, and the development of staff cultural knowledge. Potentially supportive policy mechanisms could include hiring requirements tied to community demographics, funding contingent on cultural responsiveness metrics, and technical assistance (e.g., tools to support the delivery of multi-lingual communications to parents and families) for organizations serving diverse communities. Taken together, the intervention evidence supports the core propositions of our conceptual framework. Social support networks can protect against the risks associated with adversity exposure by strengthening the protective pathways that buffer developmental challenges, and these supportive networks can be enhanced through multilevel interventions.

## Discussion

The purpose of this paper was to present an ecological model of perceived social support as a protective factor for youth exposed to adversity. Following Lynham’s (2002) Theory-Research Cycle framework for theory development, we have articulated theoretical propositions about how social support operates across ecological levels (protective mechanisms), what limits its effectiveness (barriers), and how barriers can be overcome (intervention pathways). Our conceptual model (Figure 1) proposes that perceived social support, distinct from the mere presence of supportive others, operates through a protective pathway that buffers against the risk pathway from trauma symptoms to developmental challenges. The model emphasizes three core mechanisms: (1) bidirectional relationships between trauma symptoms and perceived support, (2) multiple support sources collectively contribute to perceived support, and (3) cross-level influences such that protective mechanisms at each level are enabled or constrained by factors at higher levels (e.g., structural barriers at the macrosystem level). The next phases of theory development involve empirical testing to validate the model’s propositions, refinement based on emerging evidence, and application to practice contexts. Below, we outline critical priorities for advancing this work from conceptual development toward empirical validation and practical implementation.

### Empirical testing

Our framework proposes that trauma symptoms and perceived support interact bidirectionally, and while some empirical evidence supports this postulate (e.g., Birkeland et al., 2020), questions remain about whether and to what extent timing plays a role in this bidirectional relationship. For example, do increases in perceived support lead to decreases in trauma symptoms over time, and vice versa? Additionally, are there developmental changes that influence perceived social support at various stages? Longitudinal studies with multiple touch points could help investigate these questions.

The framework also proposes that protective mechanisms at lower ecological levels (e.g., microsystem) depend on optimal conditions at higher levels (e.g., exosystem and macrosystem). However, future studies could empirically investigate to determine if and in what ways factors at these higher levels (e.g., funding allocation) measurably influence relationship quality at the microsystem level. Furthermore, does mesosystem coordination amplify microsystem-level supports? Natural experiments could help to explore such policy changes at the macrosystem level.

Finally, there are still gaps in understanding which intervention approach effectively improves young people’s perception of social support. For example, when interventions target perceived support, which mechanisms, such as skill development or trauma-informed staff training, mediate developmental outcomes? Do higher-level interventions produce snowball effects that positively influence mechanisms and factors at other lower levels? Mediation analyses could help test our hypothesized mechanisms.

### Refinement and application considerations

There are some components of our model that should be applied in practice and further refined. The current model was developed as a foundational tool applicable across adversity types and developmental stages. However, we acknowledge that adversity type (e.g., abuse vs. poverty vs. discrimination) and the timing of exposure (early childhood vs. adolescence) likely moderate the relationships depicted (as suggested by Life Course Theory and Elder’s foundational work on children of the Great Depression, e.g., Elder et al., 2003). Future iterations should incorporate different types of adversity experiences and developmental timing as explicit moderators.

Additionally, questions remain about conditions where support alone may be insufficient. For example, youth with severe trauma symptoms may require clinical treatment before effectively perceiving available support (Cohen et al., 2014). Future research should identify these thresholds to enable more appropriate intervention matching.

Finally, to translate this model into practice, intervention protocols should be developed in partnership with practitioners within targeted communities, ensuring ecological validity and cultural responsiveness. These protocols should target each ecological level: individual-level interventions that enhance youth’s capacity to perceive support through skill-building; microsystem-level training that equips adults with trauma-informed, culturally responsive skills; coordinating efforts at the mesosystem-level that create communication across contexts; exosystem-level improvements that ensure adequate workforce capacity and access to services; and macrosystem-level policies that mandate adequate funding, appropriate staffing, and training standards.

### Limitations

Several limitations should be considered alongside the model presented in this paper. While we use directional arrows in our model, we acknowledge that much of the existing evidence is correlational rather than causal. The association between adversity exposure and trauma symptoms, while well-documented, does not definitively establish causation due to potential confounding factors (e.g., genetic vulnerabilities, pre-existing family dysfunction, and unmeasured environmental factors; Blangis et al., 2024). Therefore, the directional arrows used to represent theoretical propositions require further empirical testing through longitudinal and experimental designs.

We want to emphasize that this is a theoretical contribution rather than an empirical study; thus, the utility and accuracy of the model remain speculative. Future empirical research is needed to test and refine the model’s applicability in the field. Additionally, although we reviewed and presented both theoretical and empirical evidence that supports this model, this paper is not a scoping or systematic review and therefore does not exhaust the available literature related to this topic. As a result, it is likely that studies exist that are related to the model presented in this paper but were not included. Thus, we hope this paper is used within its scope to contribute to ongoing conversations about adversity exposures, trauma symptoms, social supports, and youth outcomes, and inspire further research on these complex factors.

## Conclusion

This conceptual paper presents a foundation for understanding how perceived social support operates as a protective factor across ecological levels. Additionally, we offer a roadmap for future empirical investigation and practical application of the conceptual model. Through rigorous empirical validation and community-partnered implementation, we hope this framework can guide efforts to strengthen support networks and promote positive development for young people navigating adversity.
